# CDK6 and p16^INK4A^ in lymphoid malignancies

**DOI:** 10.18632/oncotarget.1541

**Published:** 2013-10-24

**Authors:** Karoline Kollmann, Veronika Sexl

**Affiliations:** Institute of Pharmacology and Toxicology, University of Veterinary Medicine, Vienna, Austria; *Current affiliation: Cambridge Institute for Medical Research and Welcome Trust/MRC Stem Cell Institute and Department of Hematology; University of Cambridge; Cambridge, UK; Institute of Pharmacology and Toxicology, University of Veterinary Medicine, Vienna, Austria

Most protein kinases function as critical switches within signaling networks. Due to their central roles in signal transduction, they are subject to tight regulation and to control by both positive and negative feedback loops. The net outcome of an increase in expression and activity may be hard to predict. This has recently been shown to be the case for the cyclin-dependent kinase CDK6, which drives cells through the G1 phase of the cell cycle. Overexpression of any cyclin-dependent kinase is rather expected to promote tumour formation. However, the case of CDK6's function in lymphoma development shows that the effect may be quite the opposite.

CDK4 and CDK6 are highly homologue kinases that are considered to have largely redundant functions in driving progression from G1 to S-phase. Interestingly, whereas CDK4 is predominantly amplified in sarcoma, glioma and melanoma, high levels of CDK6 expression are generally found in haematological malignancies. In addition, a point mutation has been found in the *CDK4* gene in hereditary melanoma that prevents the protein from binding the INK4 inhibitor proteins, resulting in high kinase activity [1, 2]. To date, no mutations in human *CDK6* have been identified. It is currently unclear how deregulation of CDK4 or CDK6 is linked to distinct cancer types. One possibility is that different tissues express the two kinases at different levels. Alternatively, the kinases may have additional functions unrelated to cell cycle progression that are of importance only in certain cell types.

Our study provides initial insights into a novel and hitherto unrecognized role for CDK6 [3]. Not only is the CDK6 protein a cell cycle kinase, it is also able to act as a transcriptional regulator, a property that is not shared by CDK4. The enforced expression of CDK6 results in tumour-suppression as a consequence of the protein's transcriptional regulatory function, which incidentally is independent of its kinase activity. CDK6 has partners in crime – it cooperates with transcription factors such as STAT3 or the AP-1 member c-JUN to induce transcription of the tumour suppressor p16^INK4a^ or the angiogenic factor VEGF-A. This might seem somewhat paradoxical: VEGF-A is considered to promote tumour growth whereas p16^INK4a^ blocks tumour progression. Indeed, p16^INK4a^ may be considered as a kind of internal safeguard that protects cells from high CDK6 levels. Chromosomal translocations that involve CDK6 and result in increased expression of the gene have been reported in lymphoid malignancies. They are rare, presumably because of the protective role of the downstream tumour suppressor p16^INK4a^. p16^INK4a^ will halt and prevent excessive growth and allow the immune system to take corrective action to remove any damaged or transformed cell.

**Figure:**
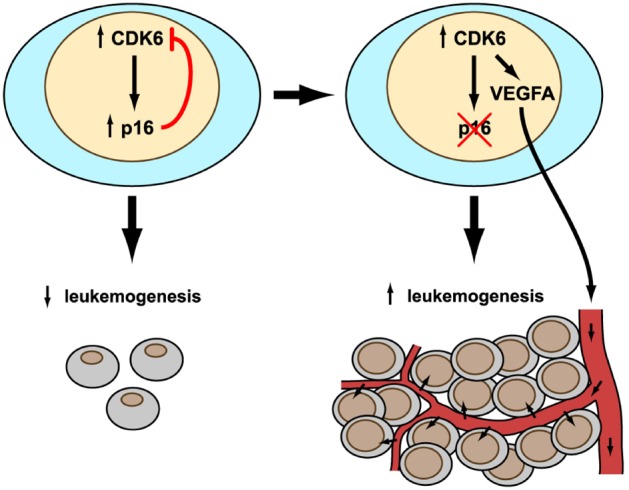


The result of CDK6 overexpression is a remarkable scenario in which the presence or absence of a single gene, *p16^INK4a^*, determines whether cell proliferation is inhibited or accelerated. Only if p16^INK4a^ is present can the feedback loop operate and normal growth be secured. If CDK6 levels are high, p16^INK4a^ will block cell cycle progression. In transformed lymphoid cells, the relative amounts of the two proteins defines the rate at which the tumour progresses. This conclusion neatly explains why CDK6 is generally overexpressed in human lymphoid malignancies while p16^INK4a^ is missing, usually due to epigenetic silencing by methylation of the promoter. Only slowly growing tumours can be kept in check by the immune system. The recent success with drugs such as PD-1 antibodies is consistent with this notion.

In summary, an evolving tumour can only derive full benefit from overexpression of CDK6 in the absence of p16^INK4a^ This reflects the situation in most human lymphoid malignancies, where CDK6 and p16^INK4a^ are expressed in an inverse manner. It is interesting that lymphomas selectively express high levels of CDK6 but not of CDK4, which could be expected to be similarly advantageous for proliferation. The selective up-regulation of CDK6 may only be understood in the light of CDK6's stimulation of VEGF-A expression. VEGF-A expression triggered by CDK6 allows tumour angiogenesis and supplies the rapidly growing malignancy with enough blood to meet its growing need for oxygen and nutrition.

The new findings have important therapeutic implications. Drugs that block CDK4/CDK6 (such as PD-0332991) are now in clinical trials but all of them are directed against the ATP-binding pocket and inhibit kinase activity. Previous studies report an enhanced sensitivity to PD-0332991 in ovarian cancer and glioblastoma cells when p16^INK4a^ expression is lost [4, 5]. This finding is consistent with p16^INK4a^'s role in modulating the effect of CDK6 overexpression. Similarly, the therapeutic advantage of combining PD-0332991 with the BCR-ABL inhibitor Imatinib is particularly high in BCR-ABL+ cell lines harbouring *p16^mK4a^* deletions. This again shows that cells with decreased or silenced p16^INK4a^ expression critically depend on CDK6. In addition, CDK6 promote tumour formation in a kinase-independent manner, for example by increasing the transcription of VEGF-A in lymphoma. We are convinced that CDK6-mediated VEGF-A expression represents the tip of the iceberg and that *VEGF-A* is only one of a number of genes regulated by CDK6 in a kinase-independent manner. In a perfect world, drugs targeting CDK6 should inhibit both its kinase activity and its kinase-independent function.
